# Nutrient Diagnosis of Fertigated “Prata” and “Cavendish” Banana (*Musa* spp.) at Plot-Scale

**DOI:** 10.3390/plants9111467

**Published:** 2020-10-30

**Authors:** Antonio João de Lima Neto, José Aridiano Lima de Deus, Vagner Alves Rodrigues Filho, William Natale, Léon E. Parent

**Affiliations:** 1 Department of Plant Science, Federal University of Ceará, Fortaleza, Ceará 60356-000, Brazil; natale@ufc.br; 2 Rural Development Institute of Paraná—IAPAR-EMATER, Curitiba, Paraná 80035-270, Brazil; aridianolima@yahoo.com.br; 3 Sítio Barreiras Fruticultura Ltd.a./Technical Sector, Missão Velha, Ceará 63200-000, Brazil; vagner@sitiobarreiras.com.br; 4 Department of Soils, Federal University of Santa Maria (visiting professor), Santa Maria, Rio Grande do Sul 97105-900, Brazil; leon-etienne.parent@fsaa.ulaval.ca; 5 Department of Soils and Agrifood Engineering, Laval University (emeritus professor), Québec City, QC G1V 0A6, Canada

**Keywords:** *clr* index, compositional data analysis, machine learning, Neural Network, perturbation vector

## Abstract

Fertigation management of banana plantations at a plot scale is expanding rapidly in Brazil. To guide nutrient management at such a small scale, genetic, environmental and managerial features should be well understood. Machine learning and compositional data analysis (CoDa) methods can measure the effects of feature combinations on banana yield and rank nutrients in the order of their limitation. Our objectives are to review ML and CoDa models for application at regional and local scales, and to customize nutrient diagnoses of fertigated banana at the plot scale. We documented 940 “Prata” and “Cavendish” plot units for tissue and soil tests, environmental and managerial features, and fruit yield. A Neural Network informed by soil tests, tissue tests and other features was the most proficient learner (AUC up to 0.827). Tissue nutrients were shown to have the greatest impact on model accuracy. Regional nutrient standards were elaborated as centered log ratio means and standard deviations of high-yield and nutritionally balanced specimens. Plot-scale diagnosis was customized using the closest successful factor-specific tissue compositions identified by the smallest Euclidean distance from the diagnosed composition using centered or isometric log ratios. Nutrient imbalance differed between regional and plot-scale diagnoses, indicating the profound influence of local factors on plant nutrition. However, plot-scale diagnoses require large, reliable datasets to customize nutrient management using ML and CoDa models.

## 1. Introduction

Brazil is the fourth largest producer of banana (*Musa* spp.) in the world, and ranks fifth in harvested area [1]. The main banana subgroups in Brazil are “Prata” (AAB), that dominates in the north and the northeast, and “Cavendish” (AAA), dominant in the south and the southeast. In 2018, the Brazilian production was 6.75 × 10^6^ Mg on 449 × 10^3^ ha, averaging 15.0 Mg ha^−1^ yr^−1^. In comparison, the average banana yield of the top ten banana producing countries reached 44.8 to 65.5 Mg ha^−1^ yr^−1^. While the productivity of banana orchards nearly doubled globally over the past 50 years from 11.7 to 20.2 Mg ha^−1^ yr^−1^, that of the Brazilian orchards stagnated around 15.0 Mg ha^−1^ yr^−1^. The frequent low productivity of Brazilian orchards is attributed to inadequate nutrient and water management [2] and insufficient soil and tissue testing [3]. Brazil is a large country which experiences highly variable rainfall regimes. In north-eastern Brazil, fertigation systems have been installed to automate water and nutrient management at the plot scale [4,5,6]. Fertilization and irrigation represent 16–22% and 14–27% of production costs, respectively [7].

Because genetic, environmental and managerial factors impact on plant elemental composition [8,9], and plants can explore the soil beyond the soil sampling layer, tissue tests are generally more closely related to crop yield than soil tests [10]. Common tissue nutrient diagnostic methods for banana crops are the “Diagnosis and Recommendation Integrated System” (DRIS) and the “Compositional Nutrient Diagnosis” (CND). Regional DRIS and CND tissue diagnostic standards have been elaborated for rainfed “Cavendish” in East Africa [11,12], irrigated “Cavendish” in Ecuador [13] and Brazil [14], and irrigated “Prata” in Brazil [15,16], using yield thresholds or boundary lines as yield separators. The latter approaches are unable to separate true negative (high yielding, nutritionally balanced) from false positive (high yielding, nutritionally imbalanced, due to luxury consumption or contamination) specimens.

Different numerical methods, cultivars and environments affect the accuracy of nutrient diagnostic standards for banana. Nevertheless, regional diagnoses are based on the assumption that all factors except those being addressed are at equal or optimum levels [17]. Regional diagnosis differs from the intuitive growers’ approach that compares unhealthy to nearby healthy specimens grown under otherwise similar conditions at a local scale. There is a need to develop a methodology to customize nutrient diagnoses of fertigated banana under similar conditions at a plot scale, where most growth-impacting factors can be assumed to be uniform.

Local scale nutrient diagnoses require collecting large amounts of high-quality data and using efficient data-processing procedures to make defective and successful compositions comparable at factor-specific levels [18]. Paradoxically, more than two hundred years ago, Alexander von Humboldt elaborated the principles of biogeography by assembling measurements, observations and local knowledge to describe complex natural systems as coherent entities including human groups [19]. It was not until recently that machine learning (ML) methods could process massive datasets to unravel complex ecosystem patterns [19,20]. ML models can predict agronomic yields from genetic, environmental and managerial features and soil and tissue tests [21,22,23].

On the other hand, Compositional Data Analysis (CoDa) methods can provide nutrient ranking in the order of their limiting effect upon yields, and report on the perturbation of soil and tissue nutrient status by fertilization. Soil and tissue nutrient concentrations are compositional data that are intrinsically multivariate and strictly positive. Such data are constrained to measurement units or scaled to the sum of fractions [24]. The CoDa methods developed to solve the closure problem in compositional data [24] confer Euclidean geometries upon soil and tissue nutrient test results, making it possible to compare compositional entities rather than separately analyzed parts. Nutritionally imbalanced compositions can thus be compared with neighboring, successful, equal-length compositions at a local scale where other yield-impacting factors are similar [18,25]. Thereafter, nutrients can be ranked in the order of their limitation to guide fertilization decisions at a plot scale, where fertigation systems can be regulated.

The ML and CoDa tools can be combined to solve nutrient problems at the plot scale in banana orchards. We hypothesized that (1) ML models could accurately predict yield from soil tests, tissue tests and local factors, and (2) local diagnoses at the plot scale, where factors interact in a unique manner, differ from regional diagnoses, where nutrient standards are averaged across factors. Our objective was to customize banana nutrition to guide fertigation decisions. Concepts of ML and CoDa are first defined to facilitate interpretation of the results at regional and local scales.

## 2. Basic Concepts to Run Nutrient Diagnoses at Plot Scale

### 2.1. Definition of Natural System and Subsystems

The notion of “system” is introduced to describe the structure of complex natural systems [26]. The objective of system analyses is to explain the successes or failures of living entities by assembling descriptive features collected as fragments of knowledge. Systems have structure and functions within boundaries. A system’s behavior depends on specific combinations of interconnected elements, traits or features. Without unifying components coherently, knowledge is merely a collection of dispersed observations, practices and conflicting perceptions, making it difficult to learn how elements are interconnected, and how to build and organize knowledge from past experience.

A cropping system can be unraveled from its quantitative and qualitative features using machine learning models [18,21]. On the other hand, a composition is a system defined explicitly by fractions of some whole, proportions or concentrations, or totaling 1, 100%, or 1000 g kg^−1^, 1,000,000 mg kg^−1^, or any other scale or measurement unit [24].

Agroecosystems are human-made ecosystems that relate living organisms to their environment within arbitrarily delineated boundaries [27] at plot, farm or territory scales [28]. The success or failure of an agroecosystem depends on many physical, biological and socio-economic factors dominated by climate, soil and management [29,30]. Present guidelines for tillage were elaborated at a territorial scale [30]. Assuming that all factors except for those being addressed are at equal or optimum levels at a territorial scale, a minimum dataset is selected to generate response curves and calibrate soil and tissue tests against crop yields in fertilizer trials [17,31], and to facilitate nutrient management [32].

The soil-plant nutrient system is most often interpreted from soil tests with respect to soil functions such as plant and biological productivity, environmental quality and animal health [33], or from tissue tests to detect internal nutrient problems [34,35,36] or enhance food quality [37]. However, many more biological, physical, chemical, ecological and cultural factors impacting plant nutrition can be combined to sustain cropping systems at local scale [27].

**Figure 1:**
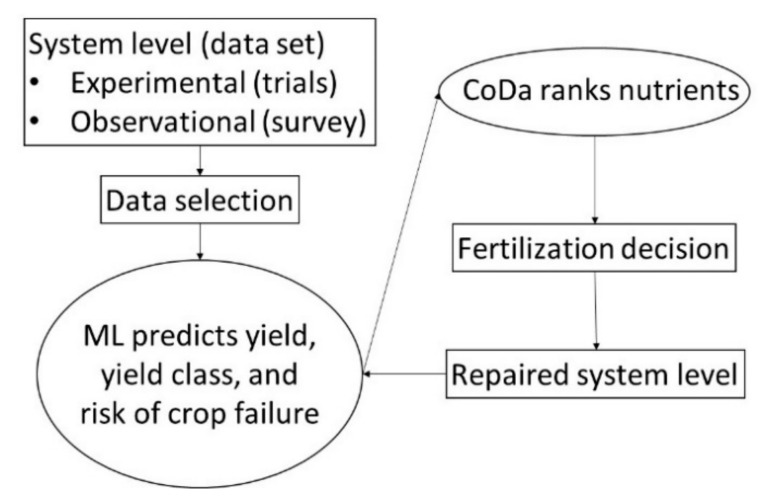
Large and diversified datasets can feed machine learning (ML) and compositional data analysis (CoDa) models to support fertilization decisions.

A great challenge is to collect large amounts of reliable, diverse experimental and observational data, and to organize the dataset for use with a model. Thereafter, data can be processed using the tools of machine learning (ML) and compositional data analysis (CoDa) to make informed fertilization decisions using a minimum dataset (Figure 1). If two ML models return the same results, the simplest one using a minimum set of data should be selected to facilitate adoption (principle of parsimony or “Occam’s razor”).

In supervised ML models, the system is defined explicitly by dependent (target) and independent (features) variables across a large number of growth-impacting factors specific to agroecosystems (Table 1). In CoDa models, the system is tied to soil and tissue tests at a plot scale, reporting analytical results (compositional data). The compositional system is closed to the unit of measurement. 

The compositional system for tissue tests is defined as simplex $S^{D}$ of *D* parts scaled on dry matter (DM) and constrained to the unit of measurement, as follows:

$$S^{D}=\left\{N,P,K,\ldots ,F_{v};N+P+K+\ldots +F_{v}=1000\;g\;kg^{-1}\right\}$$

where N, P, K, … are quantified nutrients, and $F_{v}$ is a filling value computed as follows:

$$F_{v}=1000\;g\;kg^{-1}-\sum_{i=1}^{D-1}x_{i}$$

where $x_{i}$ is the *i*th nutrient among *D*−1 nutrients in the *D*-part composition, the *D*th component being $F_{v}$. $F_{v}$ represents undertermined components such as C, H, O, …, and $S^{D}$ defines the compositional system of the diagnostic tissue on dry mass basis.

The $S^{D}$ of soil composition made of *D* parts may be defined as follows on the basis of dry mass:

$$S^{D}=\left\{sand,silt,clay,organic\;matter,\;P,\;K,\;Ca,\;Mg,\ldots ,F_{v};\sum_{i=1}^{D}x_{i}=1000,000\;mg\;kg^{-1}\right\}$$

If the system is closed to three components such as sand-silt-clay or the N-P-K relationships in plant tissue, it can be illustrated by a ternary diagram constrained to the sum of the three components. Compositional systems can also be partitioned into meaningful subsets or subsystems by combining components into orthonormal balances (see the section on isometric log ratios) [36,38,39]. Orthonormal balances provide Euclidean geometries with theoretical properties which are suitable for multivariate analyses of environmental compositional data [40].

### 2.2. Machine Learning

Supervised machine learning (ML) methods are algorithms designed to learn functional relationships in complex systems which are hidden or not defined a priori in the dataset, and to make predictions without the need to constrain assumptions regarding underlying mechanisms [41]. The dataset must be informative and requires prior knowledge on how the ecosystem under study functions. The ecosystem is defined explicitly by the categorial and continuous variables documented in the dataset and selected for modelling purposes. In contrast with traditional linear statistical models that can handle a limited number of interactions, ML algorithms address combinations of features and nonlinear relationships. The objective of ML models is to learn a function $f\left(x\right)=y$ in regression or classification mode in order to predict an output from an a priori selection of features. Most ML models have the following requirements: (1) meaningful features documented in the dataset, (2) dataset cleaned of errors and preprocessed to remove irrelevant or redundant information and avoid or reduce overfitting, and (3) the ability to predict target variables [42].

Numerous machine learning (ML) methods have been used in biology, agronomy, meteorology, bioinformatics and other disciplines. Gaussian process regression (GPR) is a Bayesian approach to regression which has been found to be appropriate to derive smooth curves from relatively small datasets such as agronomic datasets [21,22]. Other ML algorithms are artificial neural networks (ANNs), Naive Bayes, Adaboost, decision trees, Random Forests and support vector machines (SVMs) [42]. The ANN is a multilayer perceptron algorithm processing information across the input, middle and output layers of artificial neurons to optimize networking. Naive Bayes is a probabilistic classifier that assumes independence between variables. Adaboost is a boosting method to maximize the probability of a classification by reducing bias and variance. Decision trees return the probability distribution for each class given a hierarchy of features. Random Forests generate multiple decision trees to classify new instances through a voting-averaging process, making it possible to handle noise, avoid overfitting and integrate numerous features. Support vector machine separates data by maximizing the distance from a hyperplane using a kernel function. The size and diversity of the dataset and number of features selected in the model should be commensurate to avoid overfitting. The GPR, ANN and Random Forest were found to be useful ML models for small-size yet complex agronomic datasets [18,21,22,43]. Random Forests may return staircase response curves, while GPR and ANN may return smooth response curves.

### 2.3. Compositional Data Analysis (CoDa)

The CoDa paradigm was developed in the early 1980s to address problems of redundancy, scaling and spurious correlations due to the closure problem intrinsic to compositional data [24]. Compositional data are strictly positive, and multivariate data constrained between zero and the unit or scale of measurement. Due to closure, one part can be computed by determining the difference or any change in part of the whole; any change in proportion must “resonate” on others, generating redundant information, spurious correlations or absurd confidence intervals sometimes reaching beyond the limits of the compositional space (<0 or >100%). There are *D*-1 degrees of freedom in *D*-part compositions [44]. Aitchison [24] and Egozcue et al. [45] solved the closure problem using centered (*clr*) and isometric (*ilr*) log ratios possessing Euclidean geometry.

Nutrients resonating with each other within a closed compositional simplex are interconnected, and thus, behave as self-regulated systems, as opposed to a collection of parts. Nutrient interactions are traditionally expressed as dual ratios. The Brazilian literature [46] reports a plethora of physiologically meaningful dual interactions (Table 2). On the other hand, log ratios express nutrient interactions as relative values called “balances” that involve ratios and products relying on known distribution patterns. Log ratios take their origin in the logistic probability distribution function $f\left(x\right)=log\left(P/\left(1-P\right)\right)$, that is a log contrast between probability P and its additive inverse 1−P [24]. On the other hand, proportions show Dirichlet distribution, and are thus multiplicative (products).

While *clr* and *ilr* variables can be used as features in ML models, the accuracy of ML models appears to be little influenced by nutrient expressions [43]. Using raw concentrations as features may be preferable, because log ratio computation does not permit missing values, unless imputed or replaced by 0.65 times the detection limit [47], if the number of missing values is relatively small in the dataset.

#### 2.3.1. Centered Log Ratio (*clr*)

The *clr* expression is computed as follows for nutrient N as for other components [24]:

$$clr_{N}=ln\left(\frac{N}{G}\right)=ln\left(\frac{N}{{\left(N\times P\times K\times Ca\times Mg\times S\times B\times Cu\times Zn\times Fe\times Mn\times F_{v}\right)}^{\frac{1}{12}}}\right)$$

Hence,

$$clr_{N}=\frac{1}{12}ln\left(\frac{N}{N}\times \frac{N}{P}\times \frac{N}{K}\times \frac{N}{Ca}\times \frac{N}{Mg}\times \frac{N}{S}\times \frac{N}{B}\times \frac{N}{Cu}\times \frac{N}{Zn}\times \frac{N}{Fe}\times \frac{N}{Mn}\times \frac{N}{F_{v}}\right)$$

The $clr_{N}$ thus includes all possible pairwise log ratios involving N in the compositional simplex, thereby integrating most nutrients relating to N in Table 2. The *clr* method can therefore convert early attempts to address nutrient ratios such as DRIS into mathematically sound CoDa tools [48]. Note that *clr* differs from ordinary log transformation because geometric means differ between any two compositions, as follows:

$$clr_{i}-clr_{i}^{*}=ln\left(x_{i}/G\right)-ln\left(x_{i}^{*}/G^{*}\right)=ln\left(x_{i}\right)-ln\left(x_{i}^{*}\right)$$

if and only if $G=G^{*}$.

Scale-invariant centered log ratios (*clr*) can be used in biplot graphs generated in the Codapack 2.02.21 software to identify the main sources of variation in the dataset.

#### 2.3.2. Isometric Log Ratio (*ilr*)

The *ilr* variables are also called “orthonormal balances”, coordinates or Euclidean dimensions because they are orthogonal to each other. The *ilr* is computed as follows [45]:

$$ilr_{i}=\sqrt{\frac{rs}{r+s}}ln\left(\frac{G_{r}}{G_{s}}\right)$$

where *r* and *s* are numbers of parts in the numerator and denominator, respectively, and $G_{r}$ and $G_{s}$ are geometric means across those respective parts.

**Figure 2:**
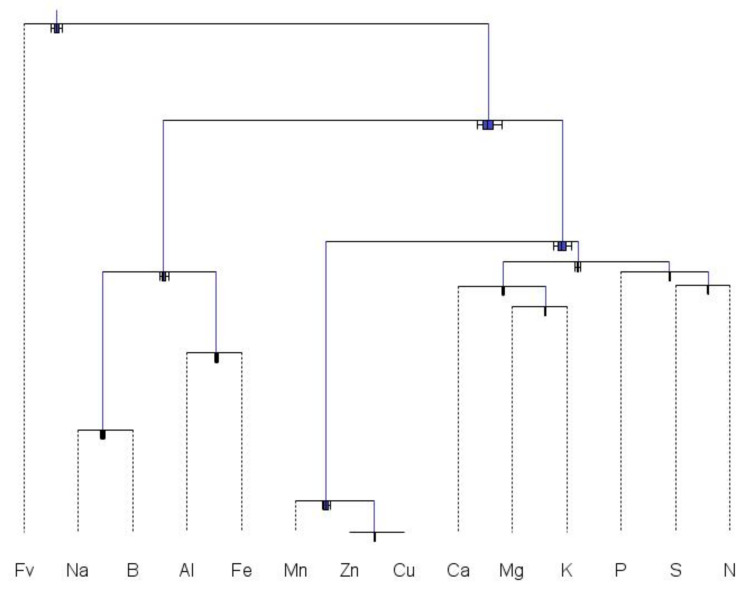
Results of tissue test arranged in a meaningful nutrient balance design using the Codapack 2.02.21 software to facilitate interpretation.

**Figure 3:**
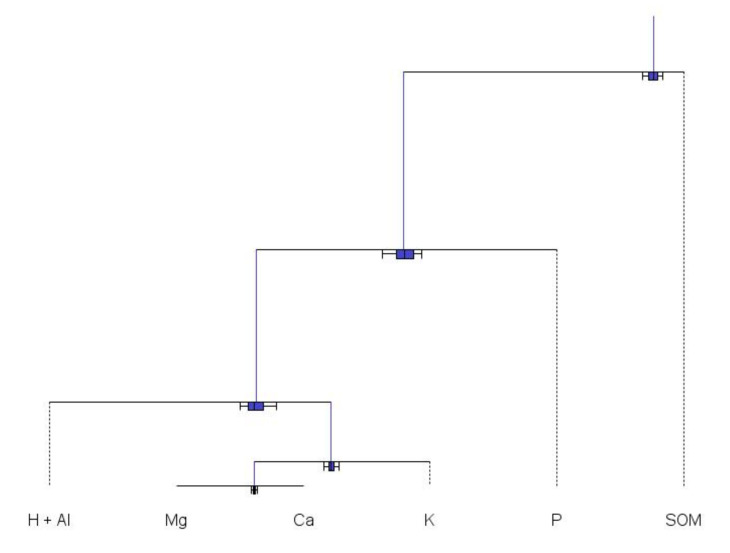
Results of soil test arranged in a meaningful nutrient balance design using the Codapack 2.02.21 software to facilitate interpretation.

Because balances are orthonormal, they return the same multivariate distance whatever their arrangement. Although there are *D*!(*D* − 1)!/2*^D^*^−1^ potential combinations of *D* *−* 1 orthonormal balances from *D*-part compositions [49], some balances are more meaningful than others in terms of interrelationships among components (e.g., nutrient interactions), for management purposes (e.g., fertilization, liming, pest management) or for appropriateness to cropping systems (soil mineralogy and alteration of minerals, quality of irrigation water). Nutrient balances can be designed as meaningful sequential binary partitions (SBPs) or balance dendrograms to facilitate interpretations of the relationships between the parts, as shown in Figure 2 for tissue tests and Figure 3 for soil tests.

In Figure 2, the first balance contrasts the filling value diluting nutrients, and the second balance contrasts components originating primarily from well water and soil mineralogy, with others. Na and B may accumulate in well water used for irrigation [50], and may thus impact plant nutrition in irrigated areas. Al and Fe may accumulate in soils as a result of alterations of soil minerals during pedogenesis [51]. Macronutrients and cationic micronutrients are contrasted to tackle N, S, P, K, Ca and Mg fertilization and Ca and Mg lime management, on the one hand, and disease control by fungicides on the other. The cationic macroelements are contrasted with N, S and P, that are generally absorbed in their anionic forms in agroecosystems [52]. K and Mg are antagonistic to each other. While the N and P ratio, also called “Redfield ratio”, reflects the balance between protein synthesis and energy transport [53], S also contributes to protein synthesis.

The results of tissue tests can also be arranged functionally according to nutrient interactions (Table 2) or phloem mobility [2]. N, P, K and Mg are mobile, S, Cu, Zn, Mn and Fe are of variable mobility, and Ca and B are relatively immobile. As an example, the balance or log contrast between concentrations of mobile and immobile nutrient can be written as follows:

$$ilr_{immobile|mobile}=\sqrt{\frac{4\times 2}{4+2}}ln\left(\frac{\sqrt[{4}]{N\times P\times K\times Mg}}{\sqrt{Ca\times B}}\right)=\sqrt{\frac{8}{6}}\left[ln\left(\sqrt[{4}]{N\times P\times K\times Mg}\right)-ln\left(\sqrt{Ca\times B}\right)\right]$$

Nutrient balances between nutrients or nutrient subsets varying in the same direction over time may also provide timelessness to the diagnosis. For example, balances computed as $\sqrt{\frac{1}{2}}ln\left(\frac{N}{P}\right)$, $\sqrt{\frac{2}{3}}ln\left(\frac{N}{\sqrt{P\times K}}\right)$ and $\sqrt{\frac{1}{2}}ln\left(\frac{\mathrm{Mg}}{\mathrm{Ca}}\right)$ may show timelessness because N, P and K concentrations in plant tissues tend to decrease, while concentrations of Mg and Ca tend to increase over time [54].

In Figure 3, soil test compositions are contrasted using first organic matter as a key property interacting with others. The dendrogram could be expanded to include soil particle-size distribution and other elements if available [55,56]. The lime requirement is described by the contrast between exchangeable cations and exchangeable acidity. An appropriate liming material is selected to rebalance soil Ca:Mg ratio.

#### 2.3.3. Euclidean Distance

The difference between the *D*-part, equal-length, diagnosed and target (*) compositions is computed as follows [24,38]:

$$\epsilon =\sqrt{\sum_{i=1}^{D}{\left(clr_{i}-clr_{i}^{*}\right)}^{2}}=\sqrt{\sum_{i=1}^{D-1}{\left(ilr_{i}-ilr_{i}^{*}\right)}^{2}}$$

### 2.4. Regional vs. Local Scales of Nutrient Diagnosis

Nutrient diagnoses at a regional scale use nutrient standards elaborated from a subpopulation of high-yielding specimens, assuming that all factors except the ones being diagnosed are at equal or optimum levels [17]. The regional CND norms are means and standard deviations of the *clr* values for true negative specimens set apart by some yield threshold in the confusion matrix. Boundary line analysis [12] or statistics [2] for parts taken in isolation may be useful to delineate nutrient standards as compatibility ranges, but may be inefficient to diagnose nutrient compositions across all parts [43]. The CDN indices are computed as follows:

$$I_{i}=\frac{\left(clr_{i}-clr_{i}^{*}\right)}{SD_{i}^{*}}$$

where $I_{i}$ is *clr* index for nutrient *i*, $clr_{i}$ is *clr* value for diagnosed specimen, and $clr_{i}^{*}$ and $SD_{i}^{*}$ are CND norms. More negative index values indicate relative nutrient shortage, while more positive index values indicate relative nutrient excess. Assuming that *clr* values are independent from each other, the CND Nutrient Imbalance Index (*CND_NII*) may be computed as follows [57]:

$$CND\_NII=\sum_{i=1}^{D}I_{i}^{2}$$

However, the *ilr* is the most appropriate transformation technique to compute multivariate distance [40]. The squared Mahalanobis distance is computed as follows:

$$M^{2}={\left(ilr-ilr^{*}\right)}^{T}COV^{-1}\left(ilr-ilr^{*}\right)$$

where *COV* is the covariance matrix and $M^{2}$ is distributed like a chisquare variable with *D*−1 degrees of freedom. M has been related to crop yield to implement the Cate-Nelson partitioning procedure [36]. The Cate-Nelson method was recently replaced by the confusion matrix generated by ML methods in classification mode [18].

On the other hand, diagnoses at a local scale avoid the cumbersome assumption at regional scales about factors other than those being diagnosed being at equal or optimal levels. Benchmark compositions for local scale nutrient diagnoses are the nutrient compositions of the closest successful specimens, i.e., the ones showing the smallest Euclidean distance using tissue and soil test results, and similarity among other features. Thereafter, nutrients can be ranked as the *clr* differences $\left(clr_{i}-clr_{i}^{*}\;\right)$ between two compositions, i.e., the diagnosed and successful ones. More negative *clr* differences indicate relative nutrient shortage. More positive *clr* differences indicate relative nutrient excess. The perturbation vector is another means to rank nutrients using the ratios between each nutrient in the diagnosed composition (X) and the corresponding nutrient in the benchmark successful composition (x), as follows [25]:

p=X⊖x=X1x1 , …, XDxD

The perturbation vector between the diagnosed and successful (*) tissue nutrient compositions is computed as follows:

$$p=\left\{\frac{N}{N^{*}},\frac{P}{P^{*}},\frac{K}{K^{*}},\ldots ,\;\frac{F_{v}}{F_{v}^{*}}\right\}$$

The perturbation vector converts into a CoDa tool the Deviation from Optimum percentage (DOP) computed as $\left(100\times C/C_{reference}\right)-100$, where C and $C_{reference}$ are diagnosed and reference nutrient concentrations, respectively [58].

## 3. Material and Methods

### 3.1. Experimental

Observational data were collected from 2010 to 2017 in 6- to 19-year-old banana stands at Missão Velha, Ceará state, Brazil (7°35′ S and 39°21′ W, 442 m in altitude). The banana dataset comprised 811 observations on cv. ‘Prata’, AAB “Prata” subgroup, and 129 observations on cv. ‘Nanica’, AAA “Cavendish” subgroup. The dataset used by Deus et al. [2] for regional scale nutrient diagnosis was augmented by adding one more year of “Prata” observations (*n* = 108 new “Prata” observations) and 129 “Cavendish” observations (2010–2017).

Soils were sandy and classified as Neossolo Quartzarênico or Quartzipsamment [51]. The regional climate is semiarid/tropical (Aw in the Köppen-Geiger classification) with dry winters and rainfall concentrated in summer. The warmest months in the area extend from September to December [59]. Maximum and minimum temperatures ranged between 31–35 °C and 19–21 °C, respectively, compared to optimal mean temperatures for banana production, i.e., 22 °C for floral initiation, 31 °C for leaf growth and development, and 28 °C (range: 15–35 °C) for high commercial yields [60]. Total rainfall averaged 1006 mm, below the 1200–1800 mm required for high fruit production.

“Prata” was planted at a density averaging 1275 plants ha^−1^ (2.8 m × 2.8 m). “Cavendish” was planted at a density averaging 1479 plants ha^−1^ (2.6 m × 2.6 m). Fertigation equipment was set to supply plant demand for water and nutrients down to plot units [7] that averaged 3.26 ha in size. Integrated pest management was carried out as recommended in [61]. Yield data were reported for both dry (July–December) and rainy (January–June) seasons. Fruit yield was reported as the sum of harvests per plot unit through the months of January to June (rainy season), and the months of July to December (dry season), respectively, and then converted to kg ha^−1^ semester^−1^.

### 3.2. Soil and Tissue Analysis

Soils and leaf tissues were analyzed as composite samples in each plot unit [62,63]. In the first and second semester every year, the third most fully expanded leaf of banana plants was collected at the blooming stage [64]. Pieces with 10-cm width were cut at the midpoint on both sides of the midrib. Four samples made of ten subsamples each were composited, then oven-dried at 72 °C and ground to less than 1 mm. The N was quantified by micro-Kjeldahl. After sample digestion in a mixture of nitric and perchloric acids [65], Ca, Mg, Fe Zn, Cu, Al and Mn were quantified by atomic absorption spectrophotometry, P and B by colorimetry, S by turbidimetry, and K and Na by emission flame photometry [66,67]. Soil samples were collected in the 0–0.20 m layer. Twenty subsamples were composited per plot into 250-cm^3^ samples, air-dried, ground and sieved to <2 mm for chemical analysis [66]. The pH was measured in 1:2.5 soil-to-water volumetric ratio. P and K were extracted using the Mehlich-1 method. Ca, Mg and Al were extracted with 1 N KCl. Elements were quantified by inductively coupled plasma (ICP-OES). Exchangeable acidity (H + Al) was extracted using calcium acetate 0.5 M at pH 7.0. Cation exchange capacity was computed as the sum of exchangeable cations (K, Ca, Mg) and exchangeable acidity. Total carbon was determined by dichromate oxidation (Walkley–Black) and multiplied by 1.724 to obtain organic matter content [68].

### 3.3. Statistical Analysis

The *clr* biplot was drawn using command Graphs-*clr* biplot in the Codapack 2.02.21 freeware. The ML classification models were run using the Orange vs. 3.23 freeware. The following machine learning (ML) models were compared in a cross-validation following classification mode: Random Forest (RF), Neural Network (NN), Naive Bayes, support vector machine (SVM), k-nearest neighbors (KNN), Adaboost and stochastic gradient decent (SGD). Model features were cultivar, year and semester of data acquisition, and tissue composition (N, P, K, Mg, Ca, S, Cu, Zn, Mn, Fe, B, Na, Al). Soil features (pH, organic matter content, available P, K, Ca, Mg, and exchangeable acidity) were used to determine the closest successful neighbors in terms of soil properties. Closeness of the successful compositional neighbors was measured as Euclidean distance. The target (dependent) variable was either fruit yield (regression mode) or fruit yield class about yield cut-off (classification mode).

Yield cut-offs between high- and low-yielders were set at 17,500 kg ha^−1^ semester^−1^ for “Prata”, and 25,000 kg ha^−1^ semester^−1^ for “Cavendish”. The confusion matrix partitioned data into true-negative (TN), false-negative (FN), true-positive (TP) and false-positive (FP) quadrants [36]. The significance of the partition was assessed by a chi-square test against equal distribution. The TN specimens were the reference subpopulation (high yield of nutritionally balanced specimens). The FN specimens presented yield limitations due to factors other than mineral nutrition. The TP specimens showed nutrient imbalance, leading to low yield. The FP specimens represented high yielders subject to luxury nutrient consumption or contamination.

Model performance was measured as area under curve (AUC) and classification accuracy (CA). The model is informative if AUC lies between 0.7 and 0.9 [69]. Classification accuracy (CA) computed as (TN + TP)/(TN + FN + TP + FP) was compared to the CA of other crops [70]. Nutrient ranges were assessed as the quartile concentration values of TN specimens. Quartiles concentration ranges of merged TN and FN specimens were reported as “compatibility intervals”, [71] indicating compatibility between the composition of the diagnosed specimen and that of the statistically reconstituted, well-balanced specimens at a regional scale [43]. The predictive model returned the probability that a diagnosed specimen would belong to the low- or the high-yielding subpopulation.

## 4. Results

### 4.1. Soil and Tissue Features

**Figure 4:**
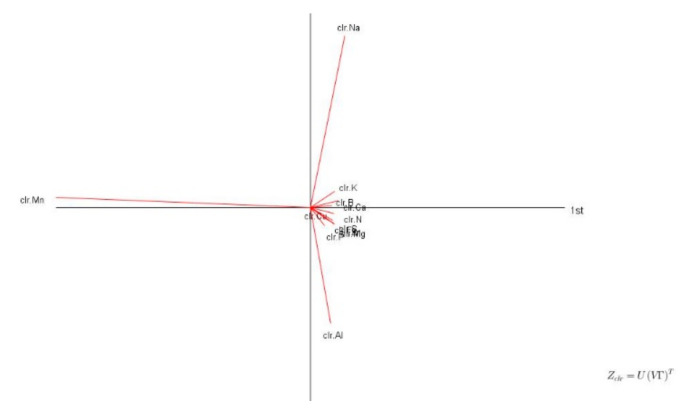
Biplot of tissue nutrient compositions of “Prata” and “Cavendish”.

Variations in soil properties and tissue compositions are presented in Table 3 and Table 4, respectively. While the means and standard deviations of soil properties generally appeared to be comparable between “Prata” and “Cavendish” plots, organic matter content, cation levels and cation exchange capacity tended to be lower in “Cavendish” plots. Biplot analysis showed that Mn, Na and Al concentrations varied the most in leaf tissues (Figure 4). The variations in tissue compositions were attributable to well water for Na, fertilization with manganese sulfate for Mn, and soil genesis or rhizosphere effects for Al [72,73].

### 4.2. Machine Learning Models

Cultivar, tissue and soil tests, field and well numbers, as well as year and semester of fruit harvests were selected as features to run ML models. Fruit yield was the target variable. The accuracy of the Neural Network (NN) and Random Forest (RF) models was higher compared to other ML models, and these models were thus retained for further analysis. Model accuracy generally increased as more information on growth-limiting factors was included (Table 5), indicating that models can learn by adding meaningful information. Cultivar alone was insufficiently informative; soil tests alone were relatively uninformative. Adding year and semester (related to climatic conditions), and plot and well numbers (related to site-specific conditions) increased accuracy markedly. The advantage of tissue tests over soil tests is that more nutrients were included as features. A tissue test alone was marginally informative. Soil and tissue tests together also returned marginal accuracy. Adding categories to tissue or soil tests, the accuracy of the NN model increased the AUC to 0.827. The partition of the confusion matrix between the TN, FN, FP and TP specimens was highly significant ($\chi^{2}=228,\;p<\;0.001$).

### 4.3. Regional Analysis

The quartile concentration ranges for nutritionally balanced TN and FN specimens are presented by cultivar across factors in Table 6. Ca was the most contrasting tissue nutrient between “Prata” and “Cavendish”. Concentration intervals were relatively narrow across all elements except Mn, Na, B and Al. The regional *clr* standards for the nutritionally balanced TN and FN specimens of “Prata” and “Cavendish” are presented in Table 7. Due to centering against the geometric mean, macronutrients (N, P, K, Mg, Ca, S) showed positive *clr* values, while micronutrients (Cu, Zn, Mn, Fe, B) and other elements (Na, Al) returned negative *clr* values. The Ca *clr* value differed markedly between “Prata” and “Cavendish”. In line with biplot analysis (Figure 4), Mn, Al and Na showed the largest variance.

### 4.4. Nutrient Diagnosis at Plot Scale

Given the median tissue concentration values in Table 4, the NN classification model predicted probabilities of 56% for diagnosed “Prata” and 44% for diagnosed “Cavendish” specimens to be classified as high yielders. Although the median concentration values were all within concentration ranges compatible with high yield potential, the “Prata” and “Cavendish” specimens still showed high probability of being imbalanced. This is because nutrient interactions and local factors were not taken into account when elaborating diagnostic standards at a regional scale.

**Figure 5:**
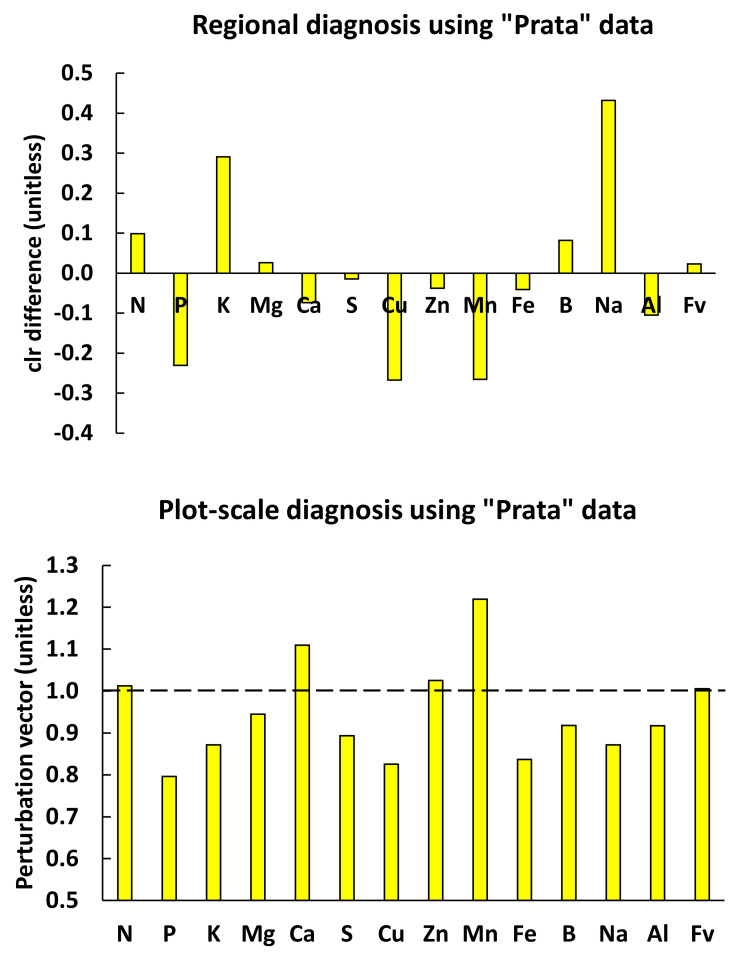
Regional diagnosis of median “Prata” tissue nutrient concentrations in Table 3 using centered log ratio index (CLR index) compared to plot-scale diagnosis using the perturbation vector. If regional and local reference compositions are the same, the *clr* differences and the perturbation vector should return similar diagnoses, which is not totally the case here, especially for N, K and Na.

**Figure 6:**
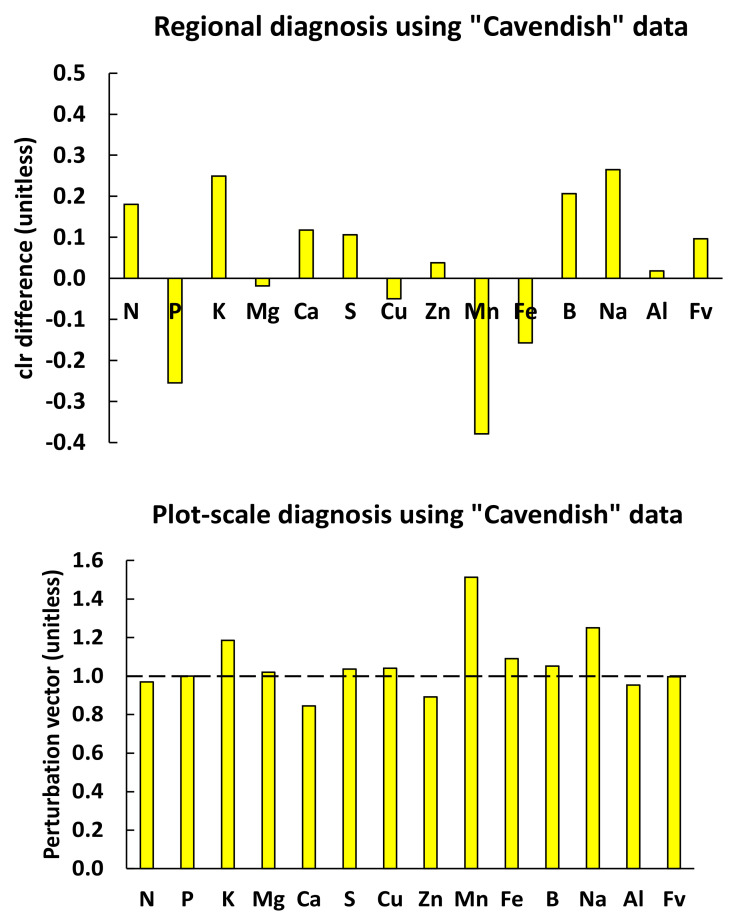
Regional diagnosis of median “Cavendish” tissue nutrient concentrations in Table 3 using centered log ratio index (CLR index) compared to plot-scale diagnosis using the perturbation vector. If regional and local reference compositions are the same, the *clr* differences and the perturbation vector should return similar diagnoses, which is not totally the case here, especially for N, Mn and Ca.

Using the nutrient standards in Table 7 computed at a regional scale, the median “Prata” tissue composition appeared to be in relative excess for N and K, and in relative shortage for P, Cu and Mn, as shown by *clr* differences (Figure 5). At the plot scale, there was a relative excess of Mg and Fe and a relative shortage of Cu and Mn, as shown by the perturbation vector. For the median “Cavendish” tissue composition in Table 7, regional diagnosis indicated a relative excess of N and K and a relative shortage of P and Mn (Figure 6); at the plot scale, the closest successful neighbor indicated a relative shortage of Ca and Zn, and a relative excess of K and Mn. Hence, regional- and plot-scale diagnoses may differ even for the same farm operation, because soil conditions and fertigation regimes may vary spatially. This is indicative of high diversity among yield-impacting factors at a plot scale, but also of the large variety of favorable factor combinations to reach high yield levels. Several successful local neighbors for diagnosed “Prata” and “Cavendish” specimens provided customized nutrient diagnoses and fertigation regimes, making it possible to reach realistic expected high yield, as documented in the dataset. The parsimonious use of nutrients and water and cost-effective decisions is supported by the proof of success of a crop located in the immediate neighborhood of the diagnosed specimen.

## 5. Discussion

### 5.1. Can Local Diagnoses Sustain the Fertigated Banana Production System?

The agroecosystem was defined from attributes impacting banana yield in relatively uniform areas to facilitate its management. On the other hand, soil samples and plant tissues were viewed as compositional systems. Subsystems were defined as associations between components and meaningful combinations of traits to meet objectives defined by the researcher or system manager. The results of soil and tissue tests could be combined into orthonormal balances to facilitate understanding nutrient relationships and compute the Euclidean distances between any two compositions.

While the nutrient requirements of banana crops are generally assessed from yield potential, nutrient exportations through harvest, restitution of plant residues back to soil, nutrient leaching, soil erosion, plantation density, soil fertility, and root development [7,72,74], there are numerous controllable and uncontrollable growth factors and countless factor interactions impacting crop performance [75]. Features can be processed by ML models at a regional scale, where yield-impacting factors are averaged, or at a local scale, where site-specific factors are documented in a dataset. Local nutrient diagnoses at the local scale are a means to adjust the banana fertigation regime to factor-specified levels. These include meteorological variables, pest management, well water quality, solum characteristics, patchy or continuous impervious layers, rockiness, gravel content, slope, aspect and other landscape patterns. Large variations in banana yield are also not only attributable to water supply, solum thickness, profile stratification, soil compaction or excessive acidity [11,76,77,78]. The root system of banana plants is concentrated in the 0–40 cm layer, and could be constrained even more by adverse soil profiles [79]. A shallow solum affects root exploitation of the soil volume. Soil limitations should be addressed by crop managers to adjust fertigation based on documented features. Some missing features are included in Table 2.

Rerunning models with additional features indicates whether such additions impact model accuracy. Removing features indicates whether a smaller set of features suffices to obtain a more parsimonious yet equally efficient model. A minimal dataset can facilitate the adoption of ML models as decision-making tools. As a routine control measure, soil tests should be conducted regularly to avoid the overaccumulation of nutrients and environmental damage, especially in intensively managed agrosystems guided by uncertain or erroneous fertilizer recommendation philosophies based on yield expectation, marginal nutrient uptake and nutrient sorption by the soil [80].

The NN predictive model indicated relatively low probability for the diagnosed median “Prata” and “Cavendish” compositions to reach high yield at a plot scale where entire compositions, not individual parts, were compared at factor-specific levels. The perturbation vector showed that several nutrients should be rebalanced by adjusting fertilizer formulations locally (Figure 5 and Figure 6). As Na and Al are not essential to the crop, any relative shortage appeared irrelevant. On the other hand, Na and Al should be checked for potential antagonisms (K-Na) or toxicity (Al) [52], especially where tissue Na and Al appear to be relatively high. Local diagnoses have advantages over regional diagnoses as guides to facilitate factor-specific fertilization decisions by changing fertilizer regimes or regional recommendations or adopting the fertilizer regime of the closest successful neighbor that also provides yield expectation.

Nutrient imbalance exposes banana plants to physiological disorders and diseases. Excessive doses of K, liming with low Mg products and irrigation with calcareous waters induce Mg deficiency, leading to a disorder called “Banana-blue” or “bleu magnésien” [60,61]. In addition, acidic soils showing high levels of Al and low levels of Ca, Mg and K are favorable to the incidence and attack by soil fungus *Fusarium oxysporum* f. sp. *cubense*, known as mal-do-panama [60,61,81]. On the other hand, higher absorption rates of basic cations than anions by banana roots produce net excretion of protons, promoting the acidification of the medium and competition among cations [73]. Protons are released to maintain the root cation/anion balance and can react with secondary clay minerals, altering the chemistry of the rhizosphere and promoting the dissolution of Al present in crystalline mineral structures, even at soil pH values exceeding 6.0 [81].

The ML, log ratio and perturbation vector can assist in adjusting banana fertigation twice a year to factor-specific conditions at the plot scale using tissue nutrient diagnoses during the blooming stage. Successful neighbors could be used as references to evaluate the adequacy of the fertigation regime over time. Local log ratio diagnoses can provide not only factor-specific fertigation, but also timelessness for crop logging during the season where climatic factors may vary. Indeed, soil moisture content varying seasonally may influence the plant nutrient uptake rate by convection and diffusion [82].

### 5.2. Need For Big Data

There are a small number of experimental data from fertilizer trials compared to the large capacity of crop managers to acquire observational data at production sites. Growers can act as citizen scientists [83,84] by providing data reliably and ethically for use with machine learning models at factor-specific levels, and to adjust fertigation locally to reach high yields with high fertilizer-use efficiency. In the present banana dataset, the fertilization regime, meteorological data, composition of well water, solum thickness, profile stratification, soil texture and soil compaction at the field level were not quantified. Meteorological data were reflected by year and semester. Nevertheless, the NN model reached an AUC of 0.827 using cultivar, tissue and soil nutrient composition, field and well numbers, and time of fruit harvest as features. Cultivar and soil tests alone contributed less than a tissue test to the model. Indeed, tissue compositions can integrate a myriad of factor interactions at the local scale. The model accuracy increased substantially by adding year, semester and plot number as proxies of meteorological conditions, well water quality and soil quality. Well water and edaphic features could provide additional site-specific yield-limiting factors to enhance model accuracy and avoid over-fertilization at the plot scale.

The concept of the “universality” of nutrient ratios [54] was rejected, even at the regional scale. In addition, regional diagnoses do not provide expected yield as the key variable currently used to make fertilizer recommendations at a factor-specific level. This implies the need to revisit the current regional diagnostic approaches to inform fertilizer recommendations for fertigated banana production. The plot is the local nutrient management unit for fertigated banana. Plot scale diagnoses evaluate growing conditions using artificial intelligence tools and compositional data analyses. Plot-scale diagnoses using successful benchmark nutrient compositions under conditions comparable to those of the diagnosed compositions provide a template for seasonal crop logging and the “timelessness” of nutrient diagnoses based on side-by-side comparisons.

It is anticipated that adding more features to support ML models and selecting minimum datasets which may easily be documented by crop managers will increase model accuracy at local scales and growers’ acceptance of the benefits of banana production and its sustainability. Nutrient standards have been documented for tissue macronutrient composition of banana crops in Africa and India [11,77], and for whole tissue composition in Brazil [2]. This paper provides a template to integrate nutrient tests and relevant genetic, environmental and managerial features into plot scale nutrient diagnoses across factor-specific Humboldtian geographical units.

## 6. Conclusions

Agroecosystems are described by specific combinations of environmental and managerial features. Compositional systems are defined explicitly by soil and tissue tests that may be arranged into balances to facilitate interpretations of the results in terms of a physiological system or for management purposes. The ML models accurately related yield to cultivar, soil and tissue tests, and other local features. The NN model, applied at a local scale, can be increasingly informed by adding more yield-impacting features, and may return nutrient diagnoses which are different than current regional diagnoses averaged across features. The NN model reached an AUC of 0.827 using cultivar, tissue and soil nutrient composition, field and well numbers, and time of harvest as features. Tissue tests that integrate all factors contributing to plant nutrition provided the most effective features to diagnose nutrient problems. This paper showed that the use of categorial features such as well or plot numbers, year and semester did not suffice to fully understand orchard performance. In particular, the state of soil and well water quality may limit the performance of fertigated banana orchards and lead to overfertilization and nutrient imbalance where yield potential is lower than average, hence reducing fertilizer-use efficiency. Fertilization dosage should thus be documented in the dataset whenever possible. Managers of banana orchards can evaluate whether regional or local diagnoses are most suitable to guide fertigation, and whether additional features must be added to improve the accuracy of predictive and recommendation models.

Using median tissue nutrient concentrations, we showed that local diagnoses at a plot scale, where factors interact, differed from regional diagnoses, where nutrient standards are elaborated across factors. While regional diagnosis provided a comparison with regional centroids weighted by their respective standard deviations for nutrient management at a regional scale, local diagnosis compared nutrient compositions as unique combinations of leaf nutrients in response to local factors. The ML-CoDa predictive models provided realistically attainable high yields at a local scale, as documented in the dataset. The site-specific probability of successfully reaching high yields can reduce uncertainty when assessing expected yield and the risk of taking erroneous fertigation decisions.

Crop managers should take part in the knowledge building process by documenting features and updating the banana dataset with observational data. Researchers can contribute to the dataset with experimental data to solve specific problems in cropping systems. Indeed, all stakeholders should collect and share data to better understand the myriad of factor interactions involved in banana production systems, and to facilitate taking informed fertilization decisions at the appropriate scale of nutrient management.

## Figures and Tables

**Table 1:** Informative datasets (features, target variables) and system boundaries used to run machine learning and compositional data analysis models on fruit production systems at a plot scale.

Method	System Closure	Feature	Target Variable
Machine learning	Plot boundary	Tissue test (diagnostic tissue): N, P, K, Mg, S, Cu, Fe, Zn, Mn, B, Na, Al, … Soil test: pH, sand, silt and clay, organic matter, P, K, Ca, Mg, exchangeable acidity, … Others: cultivar (clone), rootstock, year, semester, plot number, well number, soil classification, management practices (tillage, cover crop, training method, pest management, …), meteorological data, harvest method, …	Fruit yield Fruit yield class Nutrient offtake Fruit quality
Compositional data analysis	Measurement unit	Tissue test (diagnostic tissue): N, P, K, Mg, S, Cu, Fe, Zn, Mn, B, Na, Al, … Soil test (0–20 cm, sometimes 20–40 cm): sand, silt and clay, organic matter, P, K, Ca, Mg, exchangeable acidity, …	Euclidean distance *Clr* differences Perturbation vector

**Table 2:** Main interactions between plant nutrients [46].

Nutrient	Interaction with
N	P, K, Ca, Mg, Fe, Mn, Zn, B
P	P, K, Ca, Mg, Fe, Mn, Zn, Cu, B, Mo
K	N, P, Ca, Mg, Na, Mn, Zn, B, Mo, S, Cl
Ca	N, K, Mg, Na, Fe, Mn, Zn, Cu, B, Ni, Mo
Mg	N, P, K, Fe, Mn, Zn, B, Na, Mo, Si
S	N, P, S, Fe, Mn, Mo, B
B	N, P, K, Ca, Mg
Cu	N, P, K, Ca, Fe, Mn, Zn
Zn	N, P, K, Ca, Mg, S, Na, Fe, Zn
Fe	N, P, Ca, Mg, Cu, Mn, Zn, Mo, Ni
Mn	N, P, K, Ca, Mg, B, Mo, Ni, Zn

**Table 3:** Statistics on soil properties of fertigated plots of “Prata” and “Cavendish”.

Soil Property	“Prata” (*n* = 811 obs.)		“Cavendish” (*n* = 129 obs.)	
	Mean	SD †	Mean	SD
pH_water_	7.19	0.31	7.26	0.30
	g dm^−3^			
Organic matter content	22.0	6.9	17.8	6.5
	mg dm^−3^			
P	119.2	54.1	114.6	44.6
K	176.5	111.4	154.2	97.9
Mg	171.6	114.3	128.0	84.7
Ca	1141.2	607.1	944.2	621.3
	cmol_c_ dm^−3^			
Cation exchange capacity	8.71	4.14	7.20	4.01

† SD = standard deviation.

**Table 4:** Ranges of tissue nutrient concentrations of “Prata” and “Cavendish”.

Nutrient	“Prata” (*n* = 811 obs.)			“Cavendish” (*n* = 129 obs.)		
	Minimum	Median	Maximum	Minimum	Median	Maximum
	g kg^−1^					
N	16.4	21.9	27.0	15.2	21.9	26.3
S	0.7	1.5	7.2	1.0	1.6	2.3
P	0.7	1.6	2.9	1.1	1.6	2.2
K	13.8	34.2	59.5	22.6	37.4	51.6
Mg	1.0	2.4	4.1	1.7	2.6	3.8
Ca	1.5	6.2	11.1	1.1	8.2	14.0
	mg kg^−1^					
Cu	1.9	5.2	17.3	2.8	5.2	12.6
Zn	7.3	16.1	38.9	9.8	15.6	31.3
Mn	17.9	119.9	532.0	21.3	82.1	470.5
Fe	28.6	64.0	111.3	42.1	66.6	102.5
Al	4.1	23.3	80.2	8.7	26.7	85.3
B	1.2	10.0	30.4	2.3	10.2	21.4
Na	9.9	49.6	100.0	10.0	50.0	115.2

**Table 5:** Accuracy of machine learning classification models as more features were added.

Features	Neural Network		Random Forest	
	Area under Curve	Classification Accuracy	Area under Curve	Classification Accuracy
	From categories			
Cultivar	0.497	0.533	0.516	0.532
+year + semester + plot number	0.815	0.747	0.840	0.764
	From soil test			
Soil properties alone	0.528	0.551	0.668	0.622
+cultivar	0.534	0.547	0.667	0.618
+cultivar + year + semester + plot number	0.811	0.743	0.812	0.734
	From tissue test			
Tissue test alone	0.710	0.660	0.757	0.689
+cultivar	0.706	0.666	0.743	0.680
+cultivar + year + semester + plot number	0.827	0.750	0.784	0.691
+cultivar+year+semester +plot number + soil properties	0.820	0.746	0.802	0.729

**Table 6:** Regional quartile concentration ranges for nutritionally balanced TN and FN specimens of “Prata” and “Cavendish”.

Nutrient	“Prata” (*n* = 462 obs.)		“Cavendish” (*n* = 70 obs.)	
	Q_25_	Q_75_	Q_25_	Q_75_
	g kg^−1^			
N	20.6	22.8	20.7	22.8
S	1.4	1.6	1.4	1.6
P	1.5	1.8	1.6	1.8
K	29.3	37.0	32.0	41.6
Mg	2.2	2.7	2.4	2.9
Ca	5.7	7.1	7.1	9.4
	mg kg^−1^			
Cu	5	6	4	6
Zn	15	19	14	18
Mn	95	231	69	184
Fe	59	70	62	75
Al	18	31	22	40
B	8	13	8	12
Na	30	60	30	66

**Table 7:** Regional *clr* standards for nutritionally balanced TN and FN specimens of “Prata” and “Cavendish”.

Nutrient *clr*	“Prata” (*n* = 462 obs.)		“Cavendish” (*n* = 70 obs.)	
	Mean	SD	Mean	SD
N	3.6912	0.1306	3.6639	0.1194
S	1.0265	0.1240	1.0080	0.1162
P	1.1192	0.1389	1.0940	0.1360
K	4.0906	0.1838	4.1750	0.1952
Mg	1.4888	0.1489	1.5314	0.1582
Ca	2.4570	0.1908	2.6655	0.3049
Cu	−4.5820	0.2233	−4.6557	0.2122
Zn	−3.5007	0.1744	−3.5622	0.1874
Mn	−1.3624	0.5945	−1.6231	0.6839
Fe	−2.1283	0.1355	−2.0954	0.1345
Al	−3.1097	0.4142	−3.0136	0.4710
B	−4.0284	0.3887	−4.0743	0.3555
Na	−2.6115	0.4981	−2.5281	0.4104
Fv	7.4495	0.1214	7.4146	0.1198
